# Free tendon grafts for surgical management of chronic tears of the main body of the Achilles tendon: a systematic review

**DOI:** 10.1007/s00167-023-07446-4

**Published:** 2023-05-17

**Authors:** Nicola Maffulli, Alessandro Bartoli, Giuliano Sammaria, Filippo Migliorini, Jon Karlsson, Francesco Oliva

**Affiliations:** 1 Department of Musculoskeletal Disorders, Faculty of Medicine and Surgery, University of Salerno, 84084 Baronissi, Italy; 2 Clinica Ortopedica, Ospedale San Giovanni di Dio e Ruggi d’Aragona, 84131 Salerno, Italy; 3 Centre for Sports and Exercise Medicine, Barts and the London School of Medicine and Dentistry, Queen Mary University of London, Mile End Hospital, 275 Bancroft Road, London, E1 4DG UK; 4 School of Pharmacy and Bioengineering, Faculty of Medicine, Keele University, Thornburrow Drive, Stoke on Trent, ST4 7QB UK; 5 Department of Orthopaedic, Trauma, and Reconstructive Surgery, RWTH University Hospital, 52074 Aachen, Germany; 6 Department of Orthopaedics, Institute of Clinical Sciences, Sahlgrenska Academy, University of Gothenburg, Göteborgsvägen 31, Mölndal, 431 80 Gothenburg, Sweden

**Keywords:** Graft, Achilles tendon, Midportion, Chronic rupture, Rupture, Augmentation, Neglected

## Abstract

**Purpose:**

After four weeks from injury, tears of the Achilles tendon are considered chronic. Their management is challenging, and the use of a graft is suggested when the gap between proximal and distal stumps is greater than 6 cm. The present study systematically reviews the outcome of free tendon grafts in chronic ruptures of the Achilles tendon, evaluating clinical outcomes, complications and return to sport.

**Methods:**

The present study was conducted according to the PRISMA 2020 guidelines. PubMed, Google Scholar, Embase, and Web of Science databases were accessed in February 2023. All the published clinical studies reporting clinical outcome, return to sport and complications of free tendon grafts used the treatment of chronic rupture of the midportion of the Achilles Tendon were accessed. The mean CMS (Coleman Methodology Score) of 65.7 suggested an overall good quality of the available published articles, attesting to the low risk of bias.

**Results:**

Data from 22 articles (368 patients with a mean age of 47 years) were retrieved. The average time from rupture to surgery was 25.1 week. At last follow-up, the AOFAS (American Orthopaedic Foot and Ankle Surgery) and ATRS (Achilles Tendon Total Rupture Score) scores improved of 33.8 (P = 0.0004), and 45.1 points (P = 0.0001) respectively. Return to activity was reported in 105 patients, and 82 (78.1%) had no activity limitations, while 19 (18.1%) had limited recreational but not daily activity limitations, and 4 (3.8%) reported limitations in daily activities. Return to sport data was reported in six studies, and 45 of 93 (48.4%) patients returned to sport at an average of 22.6 weeks.

**Conclusion:**

In chronic tears of the Achilles tendon, with a gap of at least 6 cm, free tendon grafts allow predictable return to sport and acceptable recovery function.

**Level of evidence:**

Level IV.

## Introduction

The Achilles tendon (AT) is the most commonly ruptured tendon in the human body [5, 39], but at least 20% of acute ruptures are missed at first clinical examination [25]. At 4 weeks after the index injury, the rupture is considered chronic, and the tendon stumps may have retracted [33]. A palpable gap between the proximal and distal ends of the AT may have been produced, and the gap may have been at least partially filled with fibrous scar tissue [6].

Given such gap, primary repair may be not feasible [33], and different surgical procedures have been described. Generally, these are more technically demanding than primary repair, and have a greater rate of complications, including superficial infection, DVT (deep vein thrombosis), nerve injury, wound dehiscence, hypertrophic scar, and the risk of re-rupture [24].

Two classifications of chronic ruptures of the AT are reported, the Myerson classification [36] (Table 1) and the Kuwada classification [22] (Table 2). Neither is evidence based.

**Table 1:** Meyerson classification

Defect size	Treatment
Type I (rupture < 2 cm)	End-to-end anastomosis and posterior compartment fasciotomy
Type II (2–5 cm)	V–Y lengthening, augmented with tendon transfer if needed
Type III (rupture > 5 cm)	Tendon transfer alone or in combination with V–Y advancement or turndown

**Table 2:** Kuwada classification

Defect size	Treatment
Type I (partial ruptures < 50%)	Conservative management
Type II (rupture < 3 cm)	End to end anastomosis
Type III (tendinous gap 3 to 6 cm)	Often requires tendon/synthetic graft
Type IV(defect > 6 cm)	Tendon/synthetic graft and gastrocnemius recession

In chronic ruptures of the Achilles tendon with a large gap [22, 36], a local tendon transfer or a free tendon graft can be considered to restore tendon continuity [26]. In the former, the tendon of a working muscle unit around the ankle (classically, peroneus brevis, peroneus longus, flexor hallucis longus or flexor digitorum longus) is transferred to supplement the function of the gastroc-soleus complex. When using a free tendon graft, the tendon is detached from its native muscle, and used to bridge the gap between the proximal and distal stumps of the Achilles tendon [11].

Various free grafts (autograft, allografts, xenografts, synthetic grafts) have been used for this purpose [23, 28, 40], but the actual choice of one over another graft rests with the surgeon.

The present study investigates in a systematic fashion the outcome of free tendon grafts used in the management of chronic ruptures of the Achilles tendon, evaluating clinical outcomes, complications, return to activities and return to sport.

## Material and methods

### Eligibility criteria

All prospective and retrospective studies were accessed. According to the authors language capabilities, articles in English, German, Italian, and Chinese were eligible. Only clinical articles of any level of evidence according to Oxford Centre of Evidence-Based Medicine [16] were considered. Reviews, opinions, letters, editorials were not considered. Animal, biomechanics, computational, and cadaveric studies were deemed not eligible.

The PICOT algorithm was preliminarily pointed out:

- P (Problem) = Chronic rupture of the mid-portion Achilles tendon;
- I (Intervention) = Graft;
- C (Comparison) = Semitendinosus, Gracilis, Achilles, Acellular tissue tendon graft
- O (Outcomes) = Clinical outcomes, complications, and return to sport;
- T (Timing) =  ≥ 6 months of follow-up.

In February 2023, the following databases were accessed: Pubmed, Web of Science, Google Scholar, Embase. No time constrains were used for the search. Following the selection of the studies which satisfied the inclusion and exclusion criteria, we searched the reference section of each article by hand to identify other relevant investigations. The following keywords were used in combination: *Graft, Achilles tendon, midportion, chronic rupture, rupture, augmentation, neglected.*

### Search strategy

This systematic review was conducted according to the Preferred Reporting Items for Systematic Reviews and Meta-Analyses: the 2020 PRISMA statement [41]. It was registered on the International Prospective Register of Systematic Reviews (PROSPERO; Registration No. CRD42023390877).

### Selection and data collection

Two authors (A.B., G.S.) independently performed the database search. All the resulting titles were screened and if suitable, the abstract was accessed. The full text of the abstracts which matched the topic of interest were accessed. The bibliography of the full-text articles was also screened for inclusion of further articles. Disagreements were debated and the final decision was made by a third senior author (N.M.).

### Data items

Two authors independently performed data extraction. The following data were extracted: surgical technique, baseline data, aetiology of rupture, activities limitations. The primary outcome of interest was clinical outcomes. The secondary outcome of interest was return to daily activities including sports.

### Methodological quality assessment

Two authors independently performed the methodological quality assessment using the Coleman Methodology Score (CMS). The CMS is a 10-item scale designed to rate methodological quality of the included studies [4]. These items evaluated study size, mean follow-up, number of surgical approaches, type of study, diagnostic certainty, and description of surgical procedure, postoperative rehabilitation, outcome measures, outcome assessment, and selection process. The final score ranges between 0 and 100, with a score of 100 indicating the highest reported methodological quality (Tables 3 and 4).

**Table 3:** Methodological quality assessment, part A

Authors, years	Part A: only one score to be given for each of the 7 sections						
	Study size	Mean follow-up	Surgical approach	Type of study	Description of diagnosis	Descriptions of surgical technique	Description of postoperative rehabilitation
Dumbre Patil et al. 2014 [9]	4	4	10	0	5	10	5
El Shazly et al. 2014 [10]	4	4	10	10	5	10	0
Gedam et al. 2016 [13]	0	4	10	0	5	10	0
Hao et al. 2020 [14]	0	4	10	0	5	10	5
Hollawell et al. 2015 [15]	0	7	7	0	5	10	0
Jiang et al. 2019 [18]	0	4	10	10	5	10	0
Jiménez-Carrasco et al. 2023 [19]	0	10	10	0	5	10	0
Khiami et al. 2013 [20]	4	4	10	0	5	10	0
Lee et al. 2007 [23]	0	4	10	0	5	10	5
Maffulli et al. 2013 [27]	4	10	10	0	5	10	5
Maffulli et al. 2005 [28]	4	4	10	10	5	10	5
Maffulli et al. 2013 [30]	4	4	10	10	5	10	5
Nilsson et al. 2022 [37]	4	4	10	10	5	10	5
Ofili et al. 2016 [40]	0	4	10	0	5	5	0
Plaass et al. 2013 [43]	4	0	10	0	5	5	0
Qi et al. 2013[44]	0	4	10	10	5	10	5
Sarzaeem et al. 2012 [45]	0	4	10	10	5	10	0
Shoaib et al. 2017 [46]	0	4	10	10	5	5	0
Song et al. 2018 [47]	4	10	10	0	5	10	5
Tsukada et al. 2021 [48]	0	4	10	0	5	10	5
Usuelli et al. 2017 [49]	0	4	10	0	5	10	5
Vuldzhev et al. 2022 [51]	0	7	7	10	5	10	5

**Table 4:** Methodological quality assessment, Part B

Authors, years	Part B: scores may be given for each option in each of the three sections if applicable											Total
	Outcome criteria				Procedure used to assess outcomes				Description of subject selection process			
	Outcome measures clearly defined	Timing of outcome assessment clearly stated	Use of outcome criteria that have reported reliability	General health measure included	Participants recruited	Investigator independent of surgeon	Written assessment	Completion of assessment by patients themselves with minimal investigator assistance	Selection criteria reported and unbiased	Recruitment rate reported > 80%	Recruitment rate reporter < 80%	
Dumbre Patil et al. 2014 [9]	2	2	3	3	5	0	3	3	5	5	0	**69**
El Shazly et al. 2014 [10]	2	2	3	3	5	0	3	3	5	5	0	**74**
Gedam et al. 2016 [13]	2	2	3	3	5	0	3	3	5	5	0	**60**
Hao et al. 2020 [14]	2	2	3	3	5	0	3	3	5	5	0	**65**
Hollawell et al. 2015 [15]	2	2	3	3	5	0	3	3	5	5	0	**60**
Jiang et al. 2019 [18]	2	2	3	3	5	0	0	3	5	5	0	**67**
Jiménez-Carrasco et al. 2023 [19]	2	2	3	3	5	0	3	3	5	5	0	**66**
Khiami et al. 2013 [20]	2	2	3	3	0	0	3	3	5	5	0	**59**
Lee et al. 2007 [23]	2	2	3	3	5	0	3	3	0	5	0	**60**
Maffulli et al. 2013 [27]	2	3	3	3	5	0	3	3	5	0	0	**71**
Maffulli et al. 2005 [28]	2	2	3	3	5	0	3	3	5	5	0	**79**
Maffulli et al. 2013 [30]	2	3	3	3	5	0	3	0	5	5	0	**77**
Nilsson et al. 2022 [37]	2	2	3	3	5	0	3	3	5	5	0	**79**
Ofili et al. 2016 [40]	2	3	3	3	0	0	3	3	5	5	0	**51**
Plaass et al. 2013[43]	2	2	3	3	5	0	3	3	5	5	0	**55**
Qi et al. 2013 [44]	2	2	3	3	5	0	3	3	5	0	0	**70**
Sarzaeem et al. 2012 [45]	2	2	3	3	5	0	3	3	5	5	0	**71**
Shoaib et al. 2017 [46]	2	2	3	3	0	0	3	3	5	5	0	**61**
Song et al. 2018[47]	2	2	3	3	5	0	3	3	5	5	0	**75**
Tsukada et al. 2021 [48]	2	3	3	3	5	0	3	3	5	5	0	**66**
Usuelli et al. 2017 [49]	2	3	3	3	5	0	3	3	5	5	0	**66**
Vuldzhev et al. 2022 [51]	2	3	3	3	5	0	3	3	5	5	0	**76**

### Statistical analysis

The statistical analysis was performed by one author (F.M.) using the IBM SPSS version 25. Mean and standard deviation (SD) were used for descriptive statistics of continuous variables, and the frequency (events/observations) for binary data. To assess the improvement from baseline to the last follow-up of continuous variables (AOFAS, ATRS, and calf circumference), the mean difference (MD) effect measure and standard deviation were used. To investigate whether this improvement was statistically significant, the paired two tailed Student t-test was used, with values of P < 0.05 considered satisfactory.

## Results

### Study selection

**Fig. 1:**
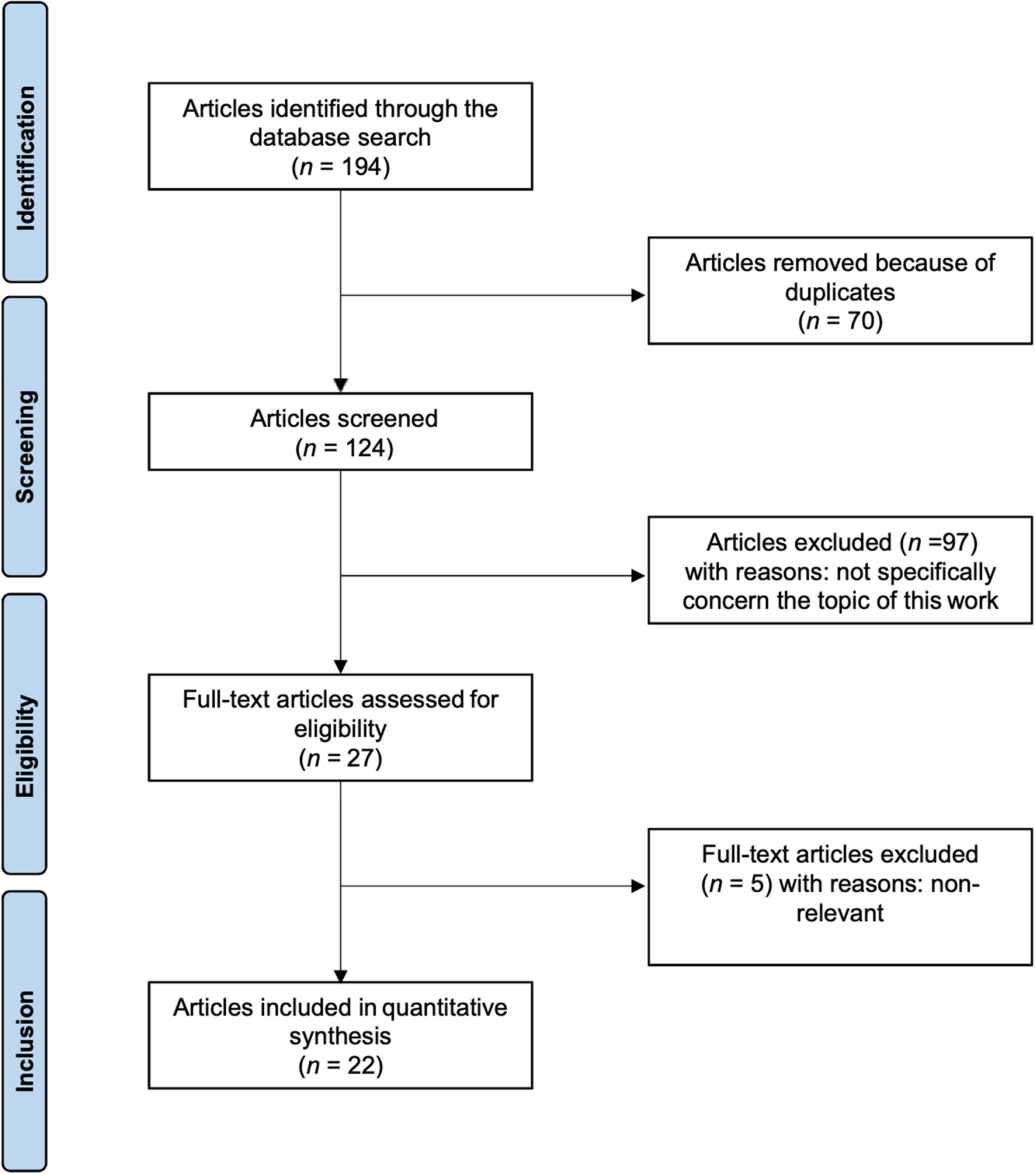
PRISMA literature search flow chart

The initial literature search resulted in 194 studies. Of them, 70 duplicates were excluded. Another 97 articles were not eligible: either not matching the topic (N = 84), focusing on surgical technique (N = 9), type of study (N = 2), full text not accessible (N = 2). This left 27 articles for inclusion. Five articles were excluded for lack of quantitative data. The articles included in the quantitative synthesis were 22: 13 retrospective and 9 prospective clinical investigations. The details of the literature search results are shown in Fig. 1.

### Study risk of bias assessment

The length of follow-up was acceptable in most studies. Surgical technique, diagnosis, and rehabilitation protocols were generally well described. The study size and the retrospective design of most of the included studies represented the main limitations highlighted by the CMS. Outcome measures, assessment timing, and selection process were also clearly defined by most studies. The mean methodology score of 65.7 suggested an overall good quality of the methodological assessment.

### Study characteristics

A total of 368 patients with a mean age of 47 years were included in the present study. A chronic tear of the Achilles tendon was diagnosed on clinical grounds in all studies and confirmed by US (ultrasound) and MRI (magnetic resonance imaging) in 14 of them. An open reconstruction was performed in 14, a mini-open in three, endoscopic surgery in three, a combination of open and mini-open surgery was reported in one study, and a combination of endoscopic and mini-open surgery was reported in one study. There was a dominance of male patients (N = 282; 76.6%) compared with females (N = 86; 23.4%). The average time from acute rupture to the index surgery was reported in 16 of 22 studies with a mean time of 25.1 weeks. In 17 of 22 studies, the distance between the Achilles tendon stumps was documented, with a mean of 6.2 cm. An autograft was used in 16 studies, four studies used allografts, one xenograft, and one a combination of allografts and xenografts. Among the 16 studies in which autografts had been used, 11 used semitendinosus only, two used a combination of semitendinosus and gracilis, one a combination of semitendinosus and FHL (flexor hallucis longus), one gracilis only, and one a free sural triceps aponeurosis. An overview of the included studies in shown in Table 5.

**Table 5:** Generalities, characteristics and outcomes of the included studies

Author et al. Year	Journal Name	Design	Graft	FU (Months)	N.P	Age	F (%)	Outcome at the last FU		
								AOFAS	ATRS	C.C. (cm)
Dumbre Patil et al. 2014 [9]	*Foot Ankle Int*	Ret	Semitendinosus autograft	30.7	35	47.4	43			
El Shazly et al. 2014 [10]	*Arthroscopy*	Pro	Semitendinosus autograft	27	15	37.7	20	90.8		
Gedam et al. 2016 [13]	*Foot Ankle Int*	Ret	Semitendinosus autograft	30.1	14	45.6	21.4	96.9	91.4	
Hao et al. 2020 [14]	*Zhonghua Wai Ke Za Zhi*	Ret	Semitendinosus + gracilis autograft	33	32	45.5	12.5	95.5	96.6	
Hollawell et al. 2015 [15]	*J Foot Ankle Surg*	Ret	Achilles and acellular tissue xenograft + allograft	37.3	4	50	0			
Jiang et al. 2019 [18]	*J Orthop Surg (Hong Kong)*	Pro	Semitendinosus + gracilis Autograft	31.3	7	47.3	14.3	97.6	92.7	
Jiménez-Carrasco et al. 2023 [19]	*J Clin Med*	Ret	Achilles allograft	82	8	45.4	33.3	95	88	
Khiami et al. 2013 [20]	*Orthop Traumatol Surg Res*	Ret	Free sural triceps aponeurosis autograft	24.5	23	52.1	13			
Lee et al. 2007 [23]	*J Foot Ankle Surg*	Ret	Acellular tissue xenograft	25	9	58.2	33	86.2		
Maffulli et al. 2013 [27]	*Am J Sports Med*	Ret	Semitendinosus autograft	98	26	42	11.5		88.0	39.7
Maffulli et al. 2005 [28]	*Clin J Sport Med*	Pro	Gracilis autograft	28.4	21	46.7	23.8		90.1	39.2
Maffulli et al. 2013[30]	*Am J Sports Med*	Pro	Semitendinosus autograft	31.4	28	46	25		86.0	37.9
Nilsson et al. 2022 [37]	*Knee aurg*	Pro	Semitendinosus autograft	12	22	60	40.9			
Ofili et al. 2016 [40]	*J Foot Ankle Surg*	Ret	Achilles allograft	16.1	14	48.4	43			
Plaass et al. 2013 [43]	*Z Orthop Unfall*	Ret	Semitendinosus autograft	3.8	26	42	11.6		88.0	
Qi et al. 2013 [44]	*Zhon. Xiu Fu Ch. Jian Wai Ke Za Zhi*	Pro	FHL + semitendinosus autograft	19.7	9	43	33.3			
Sarzaeem et al. 2012 [45]	*Knee Surg Sp Traum Arth*	Pro	Semitendinosus autograft	25	11	30	0	92.0	89.0	
Shoaib et al. 2017 [46]	*Foot Ankle Surg*	Pro	Achilles allograft	29	7	50.2	58	91.0	92.0	
Song et al. 2018 [47]	*ESSKA*	Ret	Semitendinosus allograft	53	34	36.1	11.7	100.0	99.0	
Tsukada et al. 2021 [48]	*AOFAS*	Ret	Semitendinosus autograft	35.5	10	51.7	40	95.0	86.2	
Usuelli et al. 2017 [49]	*Joints*	Ret	Semitendinosus autograft	27.9	8	50.5	37.5	92.0	87.0	37.5
Vuldzhev et al. 2022 [51]	*Cureus*	Pro	Semitendinosus autograft	30	5	47	0	92.8		38.5

*FHL* Flexor Hallucis longus, *NP* number of patients, *F* female, *Pro* prospective, *Ret* retrospective, *FU* follow-Up, *CC* calf circumference

### Synthesis of results of individual studies

At last follow-up, the AOFAS and ATRS scores improved by 37.7 (P = 0.0003) and of 51.5 (P < 0.0001) points, respectively. No statistically significant differences were found in the mean calf circumference (P = 0.1). These results are shown in greater detail in Table 6.

**Table 6:** Results of AOFAS and ATRS, and CALF circumference from baseline to the last follow-up (*MD* mean difference, *FU* follow-up)

Endpoint	Baseline	Last FU	MD	*P value*
AOFAS	56.0 ± 10.1	93.7 ± 3.7	37.7	0.0003
ATRS	38.8 ± 11.6	90.3 ± 3.9	51.1	0.0001
CALF circumference	13.6 ± 10.9	38.6 ± 0.9	10.6	0.1

Chronic midportion AT ruptures are accidents that mostly affect relatively young males (Female: 23.4%), with a mean age of 47 years.

Patient outcome was assessed both with the AOFAS scale and with the ATRS scale, with a significant improvement of both scales (Table 6).

In this systematic review, of 22 studies, 8 reported no postoperative complications [13–15, 17, 18, 47, 49, 51], one with semitendinosus autograft using an endoscopic technique, one with semitendinosus autograft using a combination of endoscopic and mini-open techniques, one using a combination of semitendinosus and gracilis using an open technique, one with semitendinosus autograft using a combination of open and mini-open techniques, one with semitendinosus autograft using a mini-open technique, one with semitendinosus allograft using an open technique, one with Achilles tendon allograft and one with a combination of allograft and xenograft using an open technique.

The most common complication was superficial post-operative infection with a prevalence of 3.5% (N = 13 of 368 patients) on the total of patients included in this review; further, wound dehiscence had a prevalence of 2.2% (N = 8 of 368 patients).

On a total of 39 post-operative complications in 368 (10.6%) patients included in this systematic review, 32 occurred in patients who had undergone reconstruction using an open technique, four a mini-open, and three an endoscopic technique (Table 7). An overview of the complications associated with the procedures is shown in Table 7.

**Table 7:** Complications (*FHL* flexor hallucis longus, *DVT* deep vein thrombosis)

Graft	Technique	Complications	Number of patients
Semitendinosus autograft [17, 43, 45, 48, 51]	Open	Hypertrophic Scar [43] Cutaneous wound problems [43] Superficial postoperative infections [45, 51] DVT [45] Sural nerve numbness [48]	1 2 2 1 1
Semitendinosus autograft [13, 27, 30, 49]	Mini-open	Persistent pain at distal wound [30] Scar adhesion to distal wound [27] Superficial postoperative infections [27]	1 1 2
Semitendinosus autograft [10, 37]	Endoscopic	Superficial postoperative infections [37] Sural nerve numbness [10, 37]	2 1
Semitendinosus + FHL autograft [44]	Open	Dehiscence [44] Injury of the tibial nerve [44]	2 1
Free sural triceps aponeurosis autograft [20]	Open	Cutaneous cicatrisation problems [20] Infracentimetric aseptic superficial skin necrosis [20] Septic partial tendon necrosis [20] Sural nerve numbness [20]	2 1 1 1
Gracilis autograft [28]	Open	Superficial post-operative infections [28]	5
Achilles tendon allograft [15, 40, 46, 47]	Open	Dehiscence [40] Superficial post-operative infections [46] Sural nerve numbness [46]	3 1 2
Acellular tissue xenograft [23]	Open	DVT [23] Dehiscence [23]	1 3

Pain was assessed in 9 studies [14, 18, 27, 28, 30, 43, 46–48], and in three, of these studies, with a mean of 8 patients, no pain [18, 46, 48] was reported. These three studies all used open techniques, with autograft (one a combination of semitendinosus and gracilis, and one semitendinosus) in 2 studies [18, 48], and with Achilles tendon allograft in the third [46]. Of the other 6 studies [14, 27, 28, 30, 43, 47], 5 used autografts (3 with semitendinosus, one with gracilis and one with a combination of semitendinosus and gracilis tendons). In the last 6 studies, semitendinosus tendon allografts were used. These 6 studies involved a total of 167 patients. One-hundred-and-forty-one (84.4%) patients reported no pain, 21 (12.6%) reported mild pain, and 5 (3.0%) moderate/severe pain. Moderate/severe pain was found using a mini-open technique with a semitendinosus autograft [27, 30], while mild pain was found in five studies. An open and a mini-open technique with semitendinosus autografts was used in one study each [27, 28, 30, 43, 47]; an endoscopic technique with a combination of semitendinosus and gracilis autograft was used in the remaining study [14] (Table 8).

**Table 8:** Characteristics of pain

GRAFT	Activity limitations (N. of patients)		
	None	limited Recreational	Daily activity
Semitendinosus autograft [10]	N = 15	N = 0	N = 0
Semitendinosus autograft [27]	N = 22	N = 2	N = 2
Gracilis autograft [28]	N = 12	N = 9	N = 0
Semitendinosus autograft [30]	N = 18	N = 8	N = 2
Achilles allograft [46]	N = 7	N = 0	N = 0
Semitendinosus autograft [49]	N = 8	N = 0	N = 0

As previously mentioned, being mostly young people or athletes, return to activity (RTA) and return to sport (RTS) are two of the major characteristics investigated, but only 6 of 20 studies addressed return to activity [10, 27, 28, 30, 46, 49] and RTS [13, 18, 20, 46, 47, 49], respectively (Tables 9 and 10).

**Table 9:** Return to activity

GRAFT	Charateristics of pain		
	None	Mild occasional	Moderate/severe
Semitendinosus autograft [14]	N = 29	N = 3	N = 0
Combination of semitendinosus and gracilis autograft [18]	N = 7	N = 0	N = 0
Semitendinosus autograft [27]	N = 20	N = 3	N = 3
Gracilis autograft [28]	N = 19	N = 2	N = 0
Semitendinosus autograft [30]	N = 20	N = 6	N = 2
Semitendinosus autograft [43]	N = 23	N = 3	N = 0
Achilles allograft [46]	N = 7	N = 0	N = 0
Semitendinosus allograft [47]	N = 30	N = 4	N = 0
Semitendinosus autograft [48]	N = 10	N = 0	N = 0

**Table 10:** Return to sport

GRAFT	Return to sports (N. patients)	Return to sport (weeks)
Semitendinosus autograft [13]	N = 1 of 14	16.0
A combination of semitendinosus and gracilis autograft [18]	N = 3 of 7	\
Free sural triceps aponeurosis autograft [20]	N = 12 of 23	30.3
Achilles allograft [46]	N = 4 of 7	16.1
Semitendinosus allograft [47]	N = 19 of 34	\
Semitendinosus autograft [49]	N = 6 of 8	28.0

Of the 105 patients in whom return to activity was documented, 82 (78.1%) reported no activity limitations, 19 (18.1%) had recreational but not daily activity limitations, and 4 (3.8%) reported limitations in activities of daily living Table 8.

RTS was documented in six studies; four using autografts (two with semitendinosus, one with a combination of semitendinosus and gracilis, one with free sural triceps aponeurosis), and two with allografts, one with an Achilles tendon allograft, and the last one with semitendinosus; 45 of 93 (48.4%) patients returned to sport at an average of 22.6 weeks following the reconstruction Table 9.

## Discussion

The most important finding of the present study based on 355 patients is that the most suitable free tendon graft to manage surgically chronic tears of the main body of Achilles tendon is probably an ipsilateral semitendinosus tendon autograft. Several free tendon grafts have been used in the management of large gaps in patients with chronic Achilles tendon ruptures, and the most commonly used tendon is an ipsilateral semitendinosus autograft.

The scales most commonly used to evaluate the clinical outcomes were the AOFAS and ATRS. Developed in 1994, the clinician-based AOFAS (American Orthopaedic Foot and Ankle Surgery) is one of the most widely used PRO (Patient-Reported Outcomes) measures for foot and ankle conditions [21]. Each measure is comprised of nine questions and covers three categories: pain (40 points), function (50 points), and alignment (10 points); 0 stands for severe pain, impairment; 100 for no pain. However, the AOFAS score has not been validated to assess the outcome of management of Achilles tendon rupture.

Nilsson-Helander et al. [38] developed the Achilles tendon Total Rupture Score (ATRS) to measure the outcome related to symptoms and physical activity after treatment in patients with total AT rupture. The scale ranged from 0 = major limitations/symptoms to 10 = no limitations/symptoms and has now been cross culturally validated and reliability tested in several languages [3, 31, 53].

Many different techniques can be used to reconstruct a chronic AT rupture, and, in general, they tend to yield similar functional outcomes. The most effective treatment for chronic Achilles rupture remains undecided, with no concrete guideline for treatment of the ATR with a defect larger than 6 cm.

Transfer of FHL or PB (peroneus brevis) are considered suitable options in chronic rupture of Achilles tendon, with good clinical outcomes and a reliable return to daily activities and sports [34].

Free tendon autograft, allograft or xenograft are described for the management of chronic rupture of the AT.

Autografts are the most commonly reported, carrying, at least theoretically, a series of advantages over other grafts:

- Healthy and strong tissue
- No disease transmission
- Fast recovery and easy harvest procedure

Hamstring tendons are commonly used as a free graft for anterior cruciate ligament (ACL) [7, 50].

These tendons are long, allowing to reconstruct the continuity of the Achilles tendon even in chronic ruptures with a wide gap between the stumps.

Wilson et al. [52] compared the load to failure, graft composite stiffness, and the elastic modulus of matched bone-patellar tendon-bone and double-looped semitendinosus-gracilis tendon grafts from young donors. The average load to failure for the patellar tendon grafts was 1784 N (± 580), significantly lower than 2 = 422 N (± 538) for the hamstring tendon grafts.

Some researchers have investigated open techniques for reconstruction of the Achilles tendon. In long-term studies, Maffulli et al. [28, 29, 32] showed that both free gracilis tendon graft and peroneus brevis tendon transfer techniques can be performed to manage, respectively, gaps up to and greater than 6 cm.

Sarzaeem et al. [45] evaluated an open technique using a free semitendinosus tendon graft to reconstruct Achilles tendon ruptures with gaps larger than 6 cm, with good functional results, providing a statistically significant improvement in terms of ATRS. Moreover, peroneus brevis tendon transfer and free gracilis tendon graft techniques [28, 29, 31, 32] lead to significant improvement in the ankle plantarflexion strength and calf circumference in the affected leg. Maffulli et al. [31, 32] showed that patients treated using a free gracilis tendon graft retained good functional results despite permanently decreased ankle plantar flexion strength and decreased calf circumference.

Allografts, on the other hand, are advantageous for their lack of donor site morbidity but they are expensive, may have limited availability, sterilization process makes tissue weaker, and a re-rupture on the same site can be a real and worrisome complication [1].

Song et al. [47] reported promising patient-reported results with low risk of re-rupture and complications utilizing semitendinosus tendon allograft for chronic ruptures of the Achilles tendon. This study reported no complications, with an ATRS of 99.0 points, with only 4 patients reporting mild pain.

Xenografts are available in great supply and wide range of sizes; however, consideration must be given to the risk of cross contamination with bovine spongiform encephalopathy or porcine endogenous retroviruses. Xenografts cannot be adequately screened for these viruses [12].

Magnussen et al. [35] reported that augmentation of Achilles tendon repair with extracellular matrix xenograft decreases gapping and increases load to failure immediately after surgery in a cadaveric model.

Lee et al. [23] showed no loss of function in augmentation of Achilles tendon using an acellular human dermal tissue matrix named GraftJacket Matrix (Wright Medical Technology Inc., Arlington, TN, USA), and favorable return to activity times without risk of re-rupture, but reported three cases of dehiscence of wound and one case of deep vein thrombosis in a small cohort of patients (N = 9).

Barrera Oro et al. [2] compared the total mean cost per case for allograft and autograft in ACL reconstruction. It was $4,147 ± $943 in the allograft group compared with $3,154 ± $704 in the autograft group (P < 0.001). Supply costs comprised a mean of 58.7% of total expenses in the autograft group and 72.2% in the allograft group. It is unclear whether similar consideration can be applied to the field at hand, thought costs are likely to be higher for allografts.

Papalia et al. [42] systematically reviewed the literature on regeneration of hamstrings after their harvest as grafts for reconstruction of the ACL. Hamstrings regeneration occurs in over 85% of operated patients as seen on histologic and imaging evaluation, but a concomitant deficit in muscular strength at deep knee flexion is most often present and remains the most concerning functional undesired effect after this procedure. Similar studies should be performed in chronic Achilles tear patients.

Although allografts are widely used in North America, in Europe there is relatively lower availability of such grafts. Also, healthcare costs limit the use of allografts in routine clinical work.

It is important that surgeons are fully conversant with several techniques depending on the patient, the local conditions, the physical requirements of the patient, and the technical abilities of the surgeons themselves.

As these procedures are not common, there could be a case for regional tertiary referral centers to offer the best available care to patients with a chronic tear of the Achilles tendon and wide retraction of the stumps which classically require these complex procedures.

Considering the average age of the patients, functional recovery is essential to return to normal daily and sports activities. The small number of patients studied does not allow to show a clear advantage of one technique over another. RTS rates are dependent on the quality of the method used to measure the RTS. To better understand RTS after chronic Achilles tendon rupture, a standardised, reliable, and valid method is required [8].

This study has several limitations. First, the retrospective design of most studies and the absence of blinding. Moreover, the inhomogeneity of the evaluation scales prevents an adequate comparison between the studies. Chronic AT ruptures especially occur in athletes, and therefore impact negatively on QoL (Quality of Life) especially in terms of return to daily activity and to sport. The present study is the first to systematically review the type of graft used in mid-portion chronic Achilles tendon rupture. However, the number of studies involved was small, and only 22 were eligible for this review. Given the lack of quantitative data available for inclusion, it was not possible to analyse the results of each graft separately.

## Conclusions

According to the main findings of the present study, when electing to use a free tendon graft the preferred graft in chronic ruptures of the midportion of the AT, with a gap about 6 cm, is semitendinosus tendon autograft.

There are no blinded studies, and no randomized control trial, and prospective investigations are few, with a strong prevalence of retrospective studies. Future studies should be planned to evaluate the superiority of given a graft over another, and whether less invasive procedures allow faster return to activities and sport with an acceptable rate of complications.

## Data Availability

The authors confirm that the data supporting the findings of this study are available within the article [and/or] its supplementary materials.
